# Regulation of matrix remodelling phenotype in gingival fibroblasts by substratum topography

**DOI:** 10.1111/jcmm.12451

**Published:** 2015-03-12

**Authors:** Shawna S Kim, Weiyan Wen, Paul Prowse, Douglas W Hamilton

**Affiliations:** a Department of Anatomy & Cell Biology, Schulich School of Medicine and Dentistry, The University of Western OntarioLondon, ON, Canada; b Division of Oral Biology, Schulich School of Medicine and Dentistry, The University of Western OntarioLondon, ON, Canada; c Graduate Program of Biomedical Engineering, Schulich School of Medicine and Dentistry, The University of Western OntarioLondon, ON, Canada

**Keywords:** dental implant, gingival fibroblasts, substratum topography, adhesion formation, integrins, titanium

## Abstract

Gingival connective tissue often has a composition resembling that of scar surrounding dental implant abutments. Increased cell adhesion, α-smooth muscle actin (α-SMA) expression and increased extracellular matrix deposition are a hallmark of fibrotic cells, but how topographic features influence gingival fibroblast adhesion and adoption of the α-SMA positive myofibroblast phenotype associated with scarring is unknown. The purpose of the present study was to demonstrate whether implant topographies that limit adhesion formation would reduce myofibroblast differentiation and extracellular matrix deposition. Human gingival fibroblasts were cultured on PT (smooth) and SLA (roughened) titanium discs for varying time-points. At 1 and 2 weeks after seeding, incorporation of α-SMA into stress-fibre bundles and fibronectin deposition was significantly higher on PT than SLA surfaces indicating differentiation of the cells towards a myofibroblast phenotype. Analysis of adhesion formation demonstrated that cells formed larger adhesions and more stable adhesions on PT, with more nascent adhesions observed on SLA. Gene expression analysis identified up-regulation of 15 genes at 24 hrs on SLA *versus* PT associated with matrix remodelling. Pharmacological inhibition of Src/FAK signalling in gingival fibroblasts on PT reduced fibronectin deposition and CCN2 expression. We conclude that topographical features that reduce focal adhesion stability could be applied to inhibit myofibroblast differentiation in gingival fibroblasts.

## Introduction

Peri-implant soft-tissue healing is critical for successful dental implant integration. The establishment of a tight seal of functional gingival connective tissue at the transmucosal region of the implant prevents downgrowth of the overlying oral epithelium 1 as well as an interface to bacterial infiltration and peri-implantitis 2. The health of the gingival margin around the abutment is therefore an important determinant of the longevity of the implants. Although dental implants are most commonly placed in a two-stage process, it is becoming increasingly common for the abutment to be inserted at the same time as the intraosseous component, making appropriate gingival attachment important in implant survival.

Gingival connective tissue healing commonly results in a tissue resembling the composition of scar tissue 3. Transforming growth factor-β (TGF-β) signalling is an important molecular regulator of healing, but also is a significant factor contributing to scar tissue formation through promotion of the α-smooth muscle actin (α-SMA) myofibroblast phenotype. Although myofibroblasts are associated with healing and secrete new extracellular matrix (ECM) in many tissues, persistence of the cells and overproduction of ECM results in scar formation 4,5. It is now known that increased adhesion and signalling through integrin β1 and focal adhesion kinase (FAK) is evident in fibroblasts in fibrosis and scarring 6. Focal adhesions (FAs) are typically peripheral sites of cell attachment containing αvβ3 integrins, but in fibrotic cells, integrin β1 is activated 4. Genetic deletion of integrin β1 prevents development of fibrotic lesions in models of skin fibrosis 7. FAK phosphorylation downstream of integrin engagement is also known to be required for the development of various types of fibrosis as it is required for TGF-β1 induction of α-SMA 8.

As alterations in substratum topography are a potent modulator of integrin expression and recruitment to adhesion sites 9–11, and FAK activation 12–14, inappropriate adhesion of gingival fibroblasts to certain topographies may be an underlying mechanism resulting in the development of fibrosis around implant surfaces. Most commercially available abutments have relatively smooth topographies, with machining marks representing the primary topography. Relatively little research has focused on how alterations in topographical features influence gingival fibroblast adhesion dynamics, downstream signalling and resulting phenotype. It has been shown that the integrin subunits expressed by human gingival fibroblasts (HGFs) at the mRNA level are not significantly altered by changes in substratum topography 15, but polished titanium (PT) topographies do increase FA number in fibroblasts compared to cells cultured on rough sand-blasted, large grit, acid-etched (SLA) topographies *in vitro* 16. We have previously shown that changes in surface roughness can reduce focal and fibrillar adhesion formation in gingival fibroblasts 17. Interestingly*, in vivo* studies have shown that these same rough SLA surfaces reduce fibrous capsule formation compared to polished topographies 18, but the molecular mechanisms underlying these observations are not understood 1,18,19. The exact relationship between altered adhesion formation in response to changes in substratum topography and downstream phenotypic changes in gingival fibroblasts is still unclear.

Gingival fibroblasts are known to exhibit a reduced adhesion capacity to ECM compared to dermal fibroblasts 20. Moreover, gingival fibroblasts have an inability to adopt a myofibroblast phenotype in response to TGF-β, which suggests an inactivation of adhesive signalling in response to TGF-β 21. Based on research showing a reduced capsule formation around SLA surfaces compared to smooth 18, we assessed whether increasing titanium surface roughness reduces α-SMA expression and increased ECM deposition through altered adhesion formation.

## Materials and methods

### Preparation of titanium surfaces

**Figure 1:**
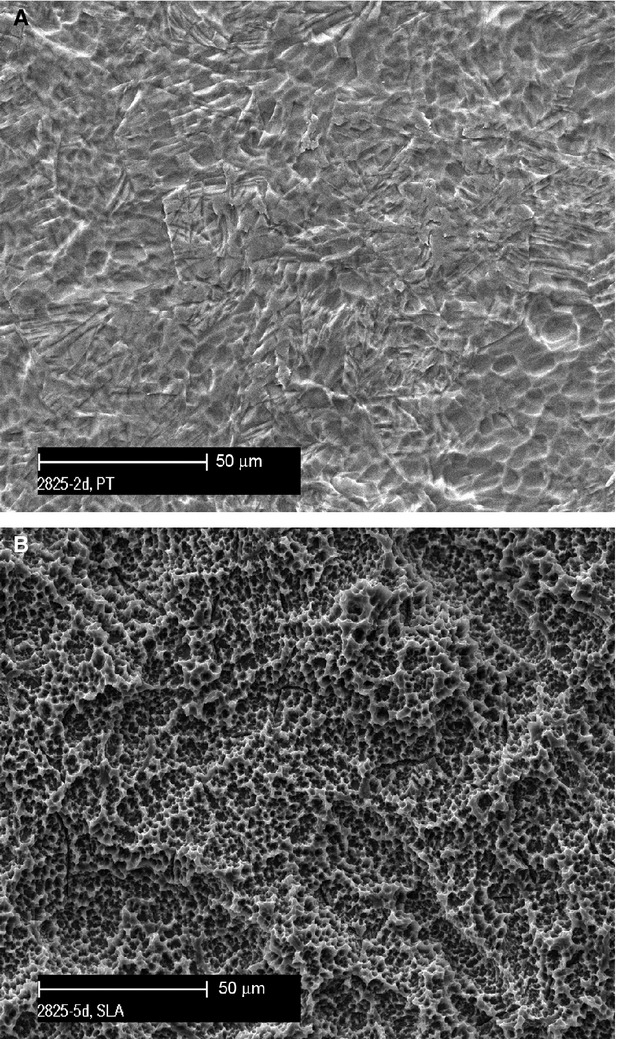
Scanning electron micrographs of the titanium topographies employed in this investigation (A) polished or PT and (B) sand-blasted, large grit, acid-etched or SLA.

PT and SLA titanium discs were prepared as previously described in Miron *et al*. 22. Briefly, PT surfaces were prepared using dilute nitric acid to clean the surface, followed by washing in reverse osmosis-purified water. SLA surfaces were prepared by blasting the titanium with corundum particles, followed by etching with HCl/H_2_SO_4_. We have previously comprehensively described the fabrication methods of each surface, as well as an analysis of the topographical features of these particular PT and SLA 22,23. The topographic features of the PT and SLA surfaces are shown in Figure1.

### HGF isolation and growth

Healthy HGFs were obtained from gingival tissue using explant cultures 24. HGFs were maintained in high glucose DMEM (Invitrogen, Carlsbad, CA, USA) supplemented with 10% fetal bovine serum (FBS; Gibco, Carlsbad, CA, USA) and 1× antibiotics and antimycotics (AA; 100 μg/ml penicillin G, 50 μg/ml gentamicin, 25 μg/ml amphotericin B), in 75 cm^2^ tissue culture plastic flasks, at 37°C in a humidified atmosphere of 95% air 5% CO_2_. Cells were removed from the growth surface with trypsin [0.25% trypsin (Gibco), 0.1% glucose, citrate-saline buffer (pH 7.8)]. Cells between passage 2 and 7 were used in experiments. HGFs were cultured on titanium discs in 24 well plates in DMEM media supplemented with 1× antibiotics/antimycotic (Invitrogen), 10% FBS (Invitrogen) and 50 μg/ml L-ascorbic acid (to facilitate collagen synthesis) (Sigma-Aldrich, St. Louis, MO, USA). To assess the effect of FAK inhibition on gene expressions, PP2 (Calbiochem, San Diego, CA, USA; 10 μm) was added to cells 15 min. prior and at the time of seeding. DMSO alone was used for control conditions. Cultures were maintained in 37°C, 5% CO_2_ incubator. Cells were seeded on titanium discs in triplicates in repeated three independent experiments for all the analysis.

### Adhesion assay

In parallel cultures, 25,000 cells/ml of HGFs were neutralized with 10 μg/ml integrin subunit specific anti-β1 (MAB2253; Millipore, Billerica, MA, USA), and anti-αvβ3 (CBL544; Millipore) and control IgG antibody for 30 min. with gentle agitation prior to culturing on titanium discs. 20,000 cells/disc was seeded for 1 hr on PT and SLA and unattached cells in the media were removed. Titanium discs were rinsed with PBS three times. HGFs were cultured on PT and SLA surfaces for 1 hr. Number of bound HGFs on surfaces were determined using CyQuant® Assay (Molecular Probes, Carlsbad, CA, USA). Experiments were done in triplicates and three independent experiments.

### Immunocytochemistry

Cells were fixed with 4% paraformaldehyde, permeabilized with 0.1% Triton X-100 and blocked with 1% bovine serum albumin (Thermo Fisher Scientific, Waltham, MA, USA). Fixed and permeabilized cells were labelled with anti-α-SMA (A5228; Sigma-Aldrich; 1:400) or mouse anti-fibronectin (sc-8422; Santa Cruz Biotechnology, Dallas, TX, USA; 1:100) which were detected with anti-mouse IgG conjugated to Alexa Fluor 488 secondary antibody (Molecular Probes). Samples were also labelled with anti-mouse vinculin (V4505; Sigma-Aldrich; 1:100) and detected with Alexa Fluor 488 conjugated secondary mouse antibody (1:200). Vinculin was double immunolabelled with rhodamine-conjugated phalloidin (Molecular Probes) for filamentous actin. To assess vinculin localization with integrin subunits β3 and β1, cells were double immunolabelled with a rabbit monoclonal antibody raised against vinculin (sc-5573; Santa Cruz Biotechnology; 1:50) and a mouse monoclonal antibody raised against integrin β3 (MAB2023Z; Millipore; 1:100), or β1 (MAB2253; Millipore; 1:100). To assess vinculin localization with tensin-1 and phosphorylated-cortactin (p-cortactin), cells were double immunolabelled with a mouse monoclonal antibody raised against vinculin, and a rabbit monoclonal antibody raised against tensin-1 (NBP1-84129; Novus Biological, Littleton, CO, USA; 1:100), or p-cortactin (05-180; Millipore; 1:100). Primary antibody binding was detected with Alexa Fluor 488-conjugated anti-mouse and anti-rabbit and rhodamine-conjugated anti-rabbit and mouse immunoglobulin (Molecular Probes). Nuclei were stained using DAPI. Images were taken on Carl Zeiss Imager M1m microscope with a dipping objective using Zen Pro 2012 software.

### Western blotting

HGFs were washed twice with PBS three times and cell lysates were harvested with RIPA buffer (Sigma-Aldrich) containing protease (Roche Diagnostics GmbH, Mannheim, Germany) and phosphatase inhibitor (Calbiochem) cocktails. Protein concentration was determined by Pierce® BCA Protein assay kit (Pierce, Waltham, MA, USA). Twenty-five microgram proteins of each sample were separated by SDS-PAGE and transferred to nitrocellulose membranes. Membranes were washed with Tris-buffered saline containing 0.05% Tween-20 (TBS-T) and blocked with 5% dried milk in TBS-T. Primary antibodies for fibronectin (sc-8422; Santa Cruz Biotechnology; 1:1000), α-SMA (A5228; Sigma-Aldrich, 1:1000) and GAPDH (MAB374; Millipore; 1:2000) were used. Fibronectin antibody used is highly reactive to matrix fibronectin and not with plasma fibronectin. Detection was with appropriate perioxidase-conjugated secondary antibodies (Jackson ImmunoResearch, West Grove, PA, USA; 1:2000), which were developed with Clarity Western ECL substrate (Bio-Rad, Hercules, CA, USA).

### RT^2^ profiler™ PCR array

Extracellular Matrix and Adhesion Molecules RT² Profiler PCR Array (PAHS-013Z; SABioscience, Frederick, MD, USA) was performed on HGFs cultured on PT and SLA for 1 and 7 days. Expressions of 84 related genes and 5 housekeeping genes were array using SYBR® Green-Based real-time PCR. The array was performed with three independent experiments using cells from three different patients. The expression levels of two genes, connective tissue growth factor (*CTGF*) and *THBS2* were confirmed using TaqMan based RT-qPCR.

### RT-qPCR

Total RNA was isolated using 1 ml of TRIzol® reagent (Invitrogen) per disc according to manufacturer's recommendations. Real-time quantitative PCR was performed on 50 ng of total RNA using TaqMan qScript™ One-Step qRT-PCR Kit (Quanta, Gaithersburg, MD, USA) and gene-specific TaqMan probes (Applied Biosystems, Carlsbad, CA, USA) under following conditions: 48°C for 30 min. followed by 90°C for 10 min. and 40 cycles of 95°C for 9 sec. and 60°C for 1 min. using 7900 Real Time PCR system (Applied Biosystems). *CTGF* and *THBS2* mRNA expressions were normalized to the housekeeping gene, *18S*. All experiments were completed in triplicates and repeated three independent experiments. PCR efficiency was verified by dilution series and relative *POSTN* mRNA level was calculated using the ΔΔCT method 25.

### Statistics

Data are expressed as the mean ± SD of three individual experiments with independent primary cultures from different participants. Individual experiments included three replicates. For quantification of FA size and myofibroblast number, 10 images per surface were analysed from three independent experiments and significance measured using Student's *t*-tests. For adhesion assay and RT-qPCR, statistical analysis was by one-way or two-way anova, as appropriate, followed by a Bonferroni correction. For RT^2^ Profiler™ PCR Array, Student's *t*-tests were performed to compare between PT and SLA at each time-points. All statistical analysis was performed with Graphpad Software v.6 (Graphpad Software, La Jolla, CA, USA; *P* ≤ 0.05 was considered significant).

## Results

### HGFs differentiate into myofibroblasts on PT

**Figure 2:**
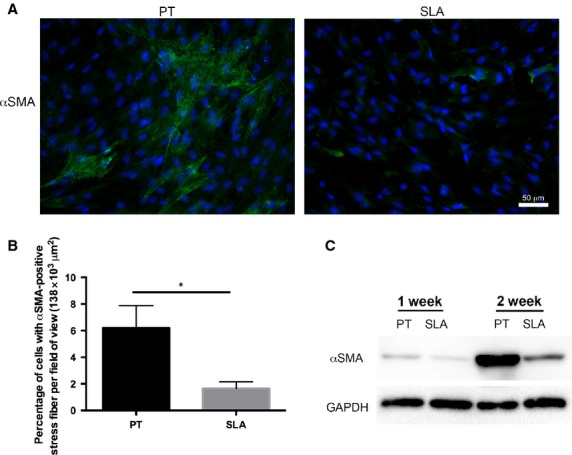
Increased differentiation of fibroblasts into myofibroblasts on PT. HGFs cultured on PT and SLA discs at 1 week. (A) Representative fluorescent images of HGFs labelled for α-SMA (green) and nuclei (blue). Myofibroblasts were revealed by α-SMA-positive stress-fibres. HGFs grew to confluence on both titanium surfaces and HGFs with α-SMA-positive stress-fibres were formed on PT. (B) Average percentage of myofibroblasts per field of view were quantified. Data represents mean ± STD of 10 images per sample with triplicate samples from three independent experiments. Data were analysed using *t*-test (*P < 0.05). Number of myofibroblasts is significantly greater on PT compared to SLA at 1 week. (C) Levels of α-SMA, a marker of myofibroblast, were assessed using western blots for HGFs cultured on PT and SLA for 1 and 2 week. GAPDH was used as a loading control.

We first assessed the level of myofibroblast differentiation on PT and SLA by fluorescently staining cells for α-SMA at 1 week. α-SMA containing stress-fibres were detected in HGFs cultured on PT and SLA (Fig.2A), with significantly fewer were present on SLA (*P* < 0.05; Fig.2B). On SLA, α-SMA was observed to be present in the cytoplasm of many cells, but was not recruited into stress-fibres. Western blotting of α-SMA demonstrated that with increasing culture time, α-SMA was higher in cells cultured on PT compared SLA at both 1 and 2 weeks after seeding (Fig.2C).

### Fibronectin fibrillogenesis

**Figure 3:**
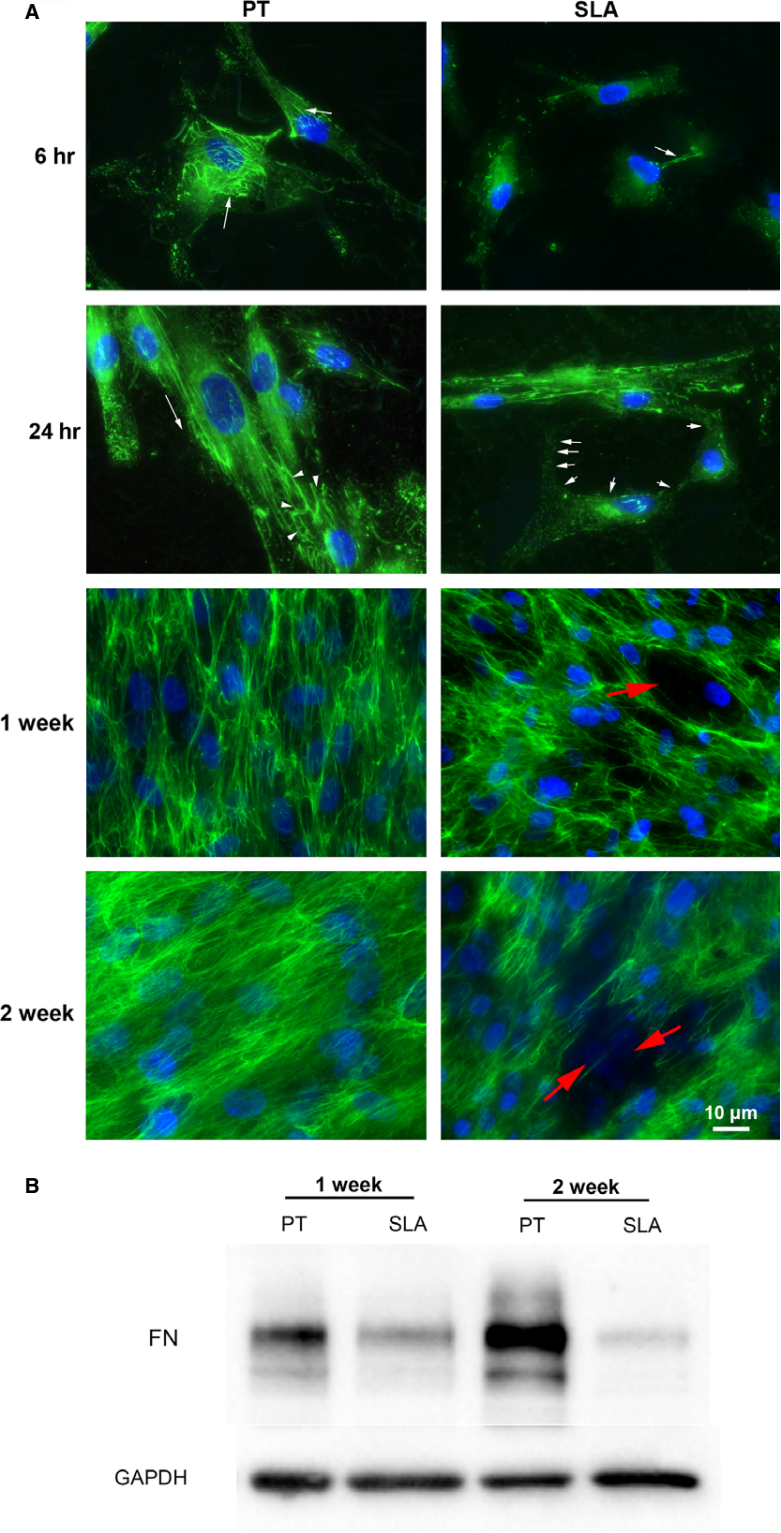
Fibronectin matrix assembly, organization and production on PT and SLA. (A) Immunocytochemical staining of HGFs on PT and SLA at 6, 24 hrs, 1 and 2 weeks. Cells were labelled for fibronectin (green) and nuclei (blue). Fibronectin fibrils (arrows) are formed within 6 hrs on PT and increases with time (arrowheads). Fibronectin is not assembled on pit holes of SLA (red arrow). (B) Western blot showing cellular and matrix fibronectin protein in cell lysates when HGFs were cultured on PT and SLA for 1 and 2 weeks. GAPDH was used as a loading control.

As myofibroblasts are known to produce excessive ECM, we next investigated whether changes in topography influenced cell fibronectin synthesis and deposition. Fibronectin fibrils developed in cells cultured on PT by 6 hrs, but were not evident in cells on SLA (Fig.3A). By 24 hrs after seeding, cells on PT continued to produce and align fibronectin. On SLA, fibril formation was evident in cells on SLA, particularly in those cells that spread around the larger pit topography caused by particle blasting of the surface. Western blot analysis of fibronectin levels in cell matrix lysate at 1 and 2 weeks after seeding demonstrated increased fibronectin protein levels in cells and matrix on PT compared to SLA at 1 week (Fig.3B).

### HGFs form smaller FAs on SLA compared to PT

**Figure 4:**
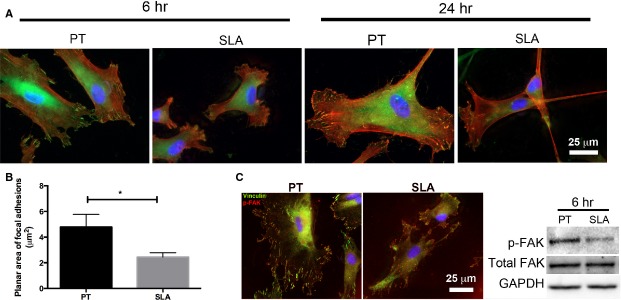
Effect of smooth and rough titanium topographies on activation of adhesive signalling. (A) Immunocytochemical staining of HGFs cultured on PT and SLA for 6 and 24 hrs. Cells were labelled for vinculin (green), phalloidin (red) and nuclei (blue). FAs were revealed by vinculin. (B) Average planar areas of FAs per cell were quantified from 10 images per topography. Data represents mean ± STD of three independent experiments in triplicates. Data was analysed using *t*-test (*P < 0.05). (C) Higher level of p-FAK in HGFs on PT compared to SLA. Immunocytochemical staining of HGFs cultured for 6 hrs on titanium discs. Cells are labelled for p-FAK (red), vinculin (green) and nuclei (blue). Levels of p-FAK in HGFs on PT and SLA are assessed using western blotting. Total-FAK and GAPDH are used as loading controls.

To assess influence of surface topography on FA formation, HGFs were cultured on titanium discs and FA distribution and size were analysed using immunofluorescence staining of vinculin. On PT at 6 hrs, vinculin was localized at the periphery of the cells at the ends of filopodia and stress-fibres (Fig.4A). On SLA, vinculin was faintly detected at the periphery at 6 hrs. On PT at 24 hrs, increased number of vinculin-containing focal complexes was found at the ends of stress-fibres, in the lamellopodium and lamellae. Small punctate focal complexes started to be evident on SLA at 24 hrs. Average planar area of FAs formed at 24 hrs was significantly greater on PT compared to those on SLA (*P* < 0.05; Fig.4B). FAK is a critical regulator of integrin-mediated adhesive signalling. FAK activation in HGFs cultured on PT and SLA for 6 hrs were assessed using immunofluorescence for distribution of p-FAK and western blotting for level of p-FAK. On PT, we observed that p-FAK was co-localized with vinculin-containing FAs (Fig.4C). On SLA, p-FAK was also localized in previously observed-smaller FAs and sizes of p-FAK staining were also smaller. Level of FAK phosphorylation at 6 hrs assessed using western blotting demonstrated that p-FAK was higher on PT compared to SLA (Fig.4C).

### Recruitment of integrin subunits to FAs on PT and SLA

**Figure 5:**
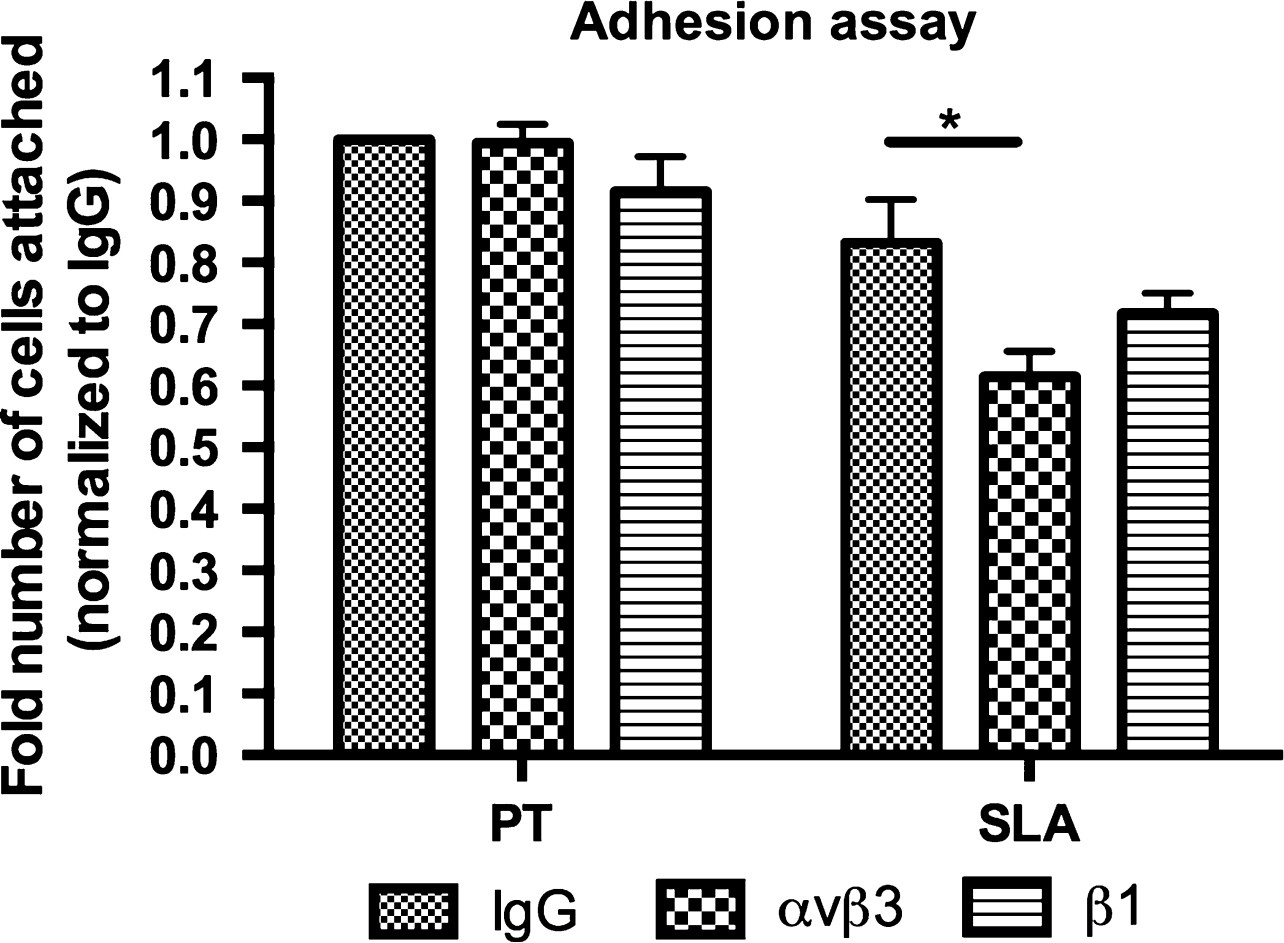
Influence of specific integrin subunit neutralization on HGF adhesion on PT and SLA. Integrin αvβ3, and β1 of HGFs were neutralized using the integrin subunit specific antibodies. Data represents relative number of attached cells after 1 hr of seeding and error bars represent STD. Data were analysed using two-way anova with bonferonni after test (*P < 0.05).

**Figure 6:**
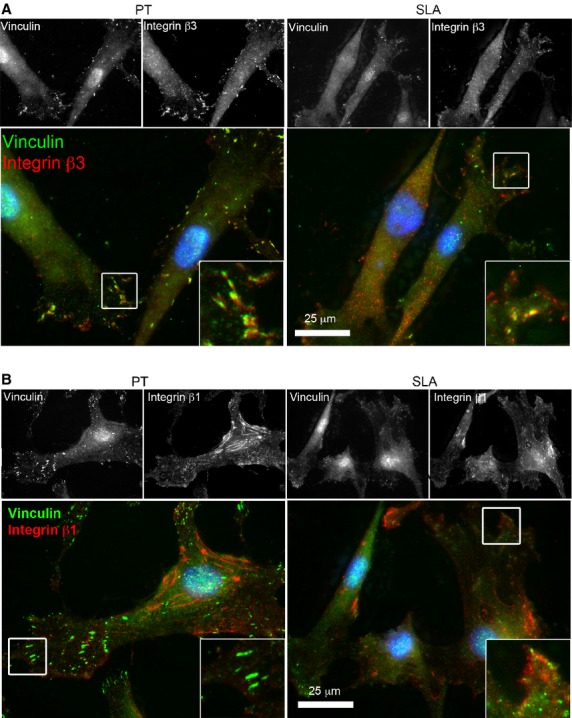
Co-localization of integrin β3 and β1 with vinculin in HGFs on PT and SLA titanium surfaces. Immunocytochemical staining of HGFs cultured on PT and SLA surfaces at 24 hrs. Cells were labelled for integrin β3 (red) (A) or integrin β1 (red) (B), vinculin (green) and nuclei (blue). Representative images of HGFs on PT and SLA observed under fluorescence microscopy. Grey scale images are shown for separate channels for integrin subunits and vinculin. Inserts show higher magnifications of focal contacts (white boxes).

In normal cells, αvβ3 is the predominant integrin recruited to sites of adhesion development. However, in fibrotic cells β1 is instead recruited. We first examined whether alterations in topography influenced the specificity of integrin subunits recruited to adhesion sites. Neutralization of αvβ3, but not β1 led to significant reduction in HGF adhesion compared to control IgG neutralized cells on SLA (*P* < 0.05; Fig.5). Neutralizing integrin subunits αvβ3 or β1 did not significantly change cell attachment on PT. To confirm the type of integrin subunits in FAs on each surface, integrin β3 or integrin β1 was co-localized with vinculin (Fig.6A and B). On PT, integrin β3 was co-localized with vinculin, but on SLA, clusters of integrin β3 were evident that did not co-localize with vinculin (Fig.6A). Co-localization of integrin β1 on PT and SLA demonstrated that very few FAs contained integrin β1, with most labelling for β1 in the central area of the cells where fibrillar adhesions form (Fig.6B).

### More mature and stable adhesions form on PT

**Figure 7:**
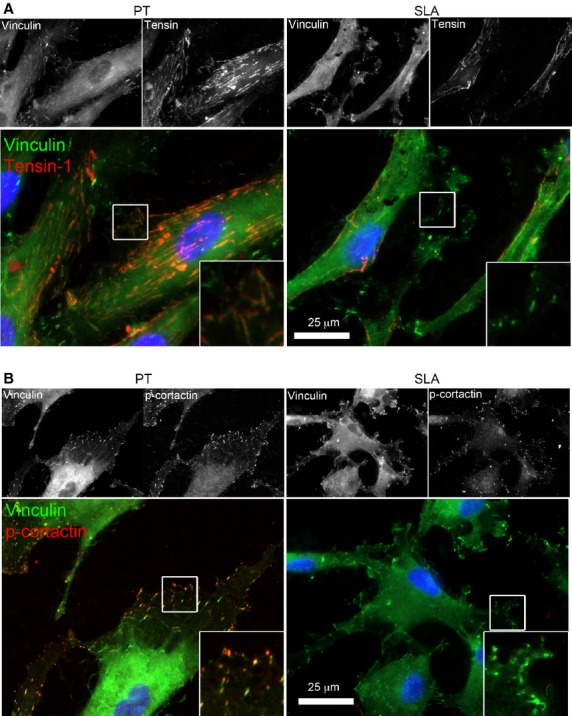
Adhesions are more mature and stable on PT compared to SLA. Co-localizations of tensin-1 and p-cortactin with vinculin were shown by immunocytochemical staining of HGFs cultured on PT and SLA surfaces at 24 hrs. Cells were labelled for integrin tensin-1 (red) (A) or p-cortactin (red) (B), vinculin (green), and nuclei (blue). Representative images of HGFs on PT and SLA observed under fluorescence microscopy. Grey scale images are shown for separate channels for tensin-1, p-cortactin and vinculin. Inserts show higher magnifications of focal contacts (white boxes).

As FAs mature into fibrillar adhesions, other proteins are recruited including vinculin and tensin 26,27. To assess maturity and composition of FAs, double immunofluorescence was performed on HGFs cultured for 24 hrs for vinculin with either tensin-1 or phosphorylated-cortactin (p-cortactin). Level and distribution of tensin was assessed by immunofluorescence at 24 hrs. Tensin-1 was identified in fibrillar adhesions in plaques parallel to the longitudinal direction of the cell shape on both PT and SLA (Fig.7A), although at a greater level on PT. Co-localization of tensin-1 and vinculin was also assessed for mature FAs. Inserts showing higher magnification demonstrate that tensin-1 and vinculin co-localized on PT but not on SLA. P-cortactin and vinculin co-localization was assessed next. Vinculin-containing FAs were co-localized with p-cortactin on PT at 24 hrs (Fig.7B). Some of p-cortactin plaques were present in the leading edges of lamellipodium without vinculin co-localization on PT. This suggests that p-cortactin may be recruited to both nascent and FAs on PT. On SLA, the intensity and size of p-cortactin staining was relatively weak and they were not co-localized with vinculin.

### SLA induces expression of genes associated with ECM remodelling

**Figure 8:**
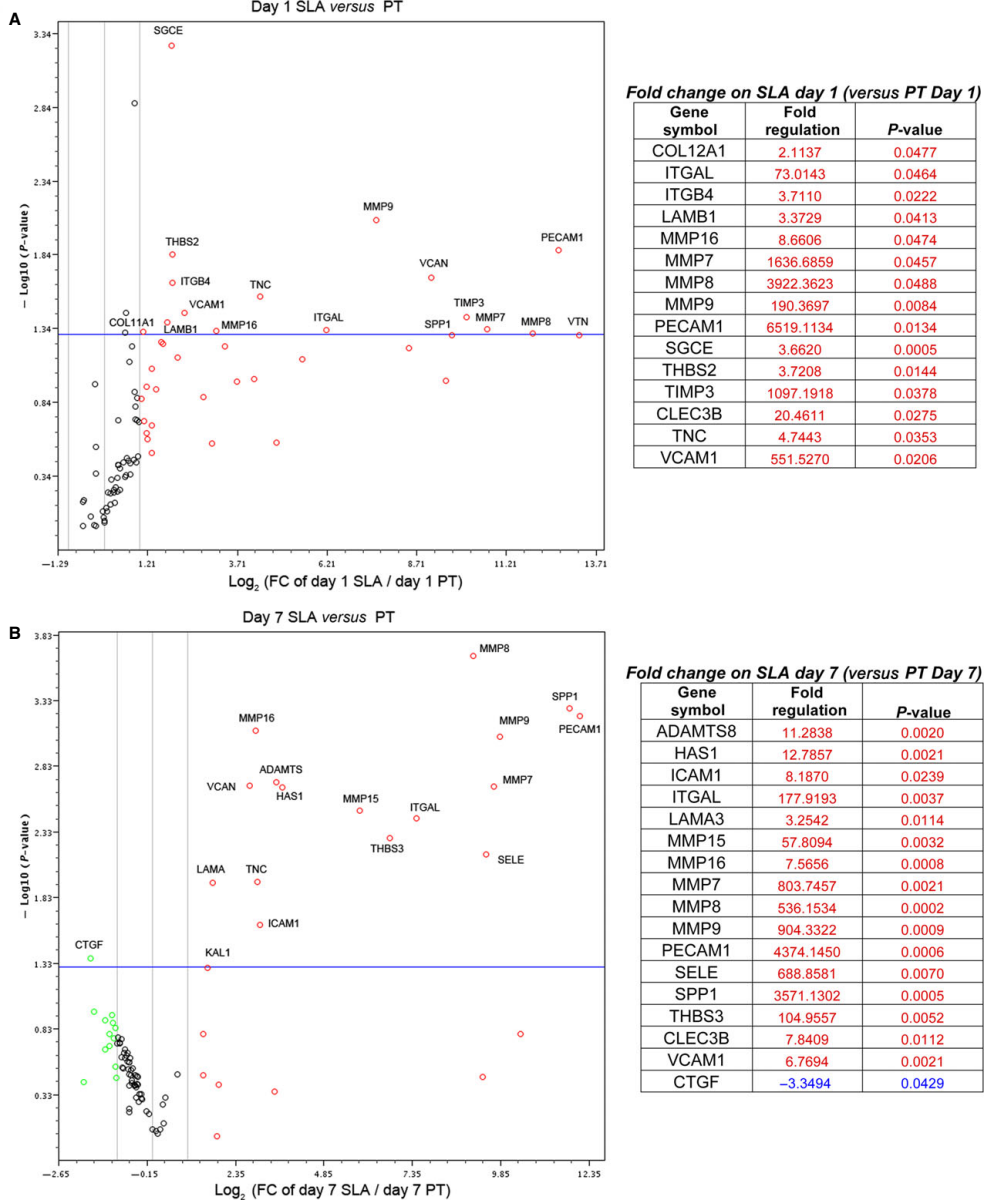
Differential expression of Extracellular Matrix and Adhesion Molecule genes on PT and SLA. HGFs cultured on PT and SLA for 1 day (A) and 7 days (B) were subjected to Extracellular Matrix and Adhesion Molecules RT² Profiler™ PCR Array. Gene expression is represented in volcano plots. *X*-axis represents log of the fold change on SLA compared to PT. *Y*-axis represents negative log of the *P*-values, respectively. Vertical lines represent twofold cut-off on SLA compared to PT. The Horizontal line shows where *P* = 0.05 with points above the line having *P* < 0.05. Each dot represents mean of three replicates. Genes significantly changed in the plot are shown in the listed with fold change and *P*-values. Gene expressions of molecules involved in tissue remodelling were significantly up-regulated on SLA compared PT. Connective tissue growth factor, known to induce fibrosis, was down-regulated on SLA compared to PT.

During tissue development and remodelling, events involving ECM and adhesion molecules are activated. We investigated whether the titanium topography influences gene expressions of ECM and adhesion molecules of HGFs cultured on PT and SLA by performing ECM and Adhesion molecules RT^2^ Profiler™ PCR Array (Fig. S1). Of the 84 tested genes, 15 and 16 genes increases at 1 and 7 days respectively, and only 1 gene decreased at 7 days on SLA compared to PT (Fig.8A and B). Several matrix metalloproteinases (MMPs) were expressed significantly higher on SLA compared to PT. Greater than 1000-fold increases in *MMP-7, 8* and *9, PECAM-1,* and tissue inhibitors of metalloproteinase 3 (*TIMP3*) mRNA levels on SLA were detected at both 1 and 7 days. The fibrotic mediator *CTGF* was the only gene that was significantly lower on SLA at 7 days (*P* < 0.05; Fig.8B).

### FAK inhibition attenuates increased *CTGF* and augments decreased *THBS2* on PT

**Figure 9:**
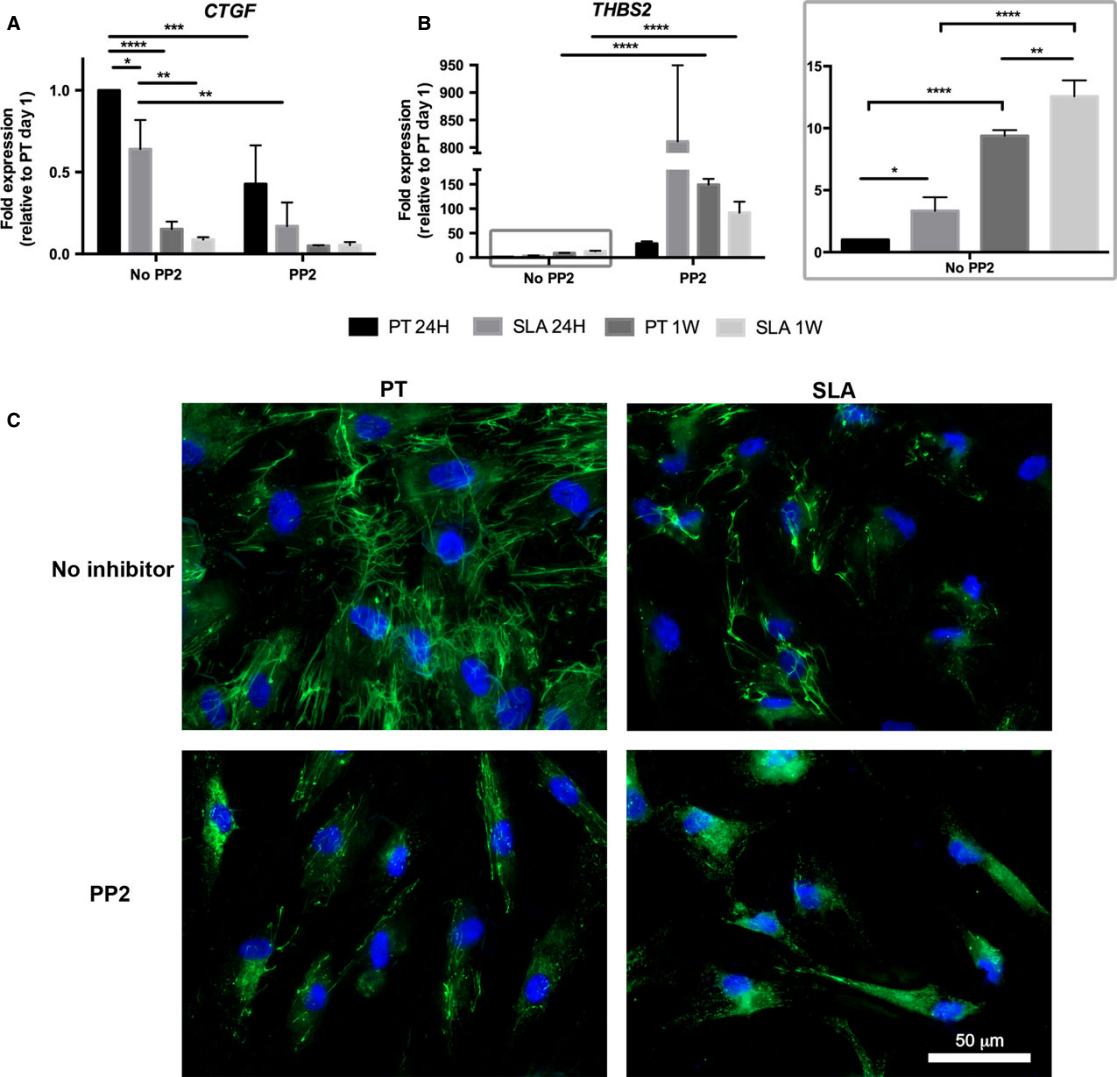
Effect of FAK inhibition on matrix protein production. (A and B) HGFs cultured on PT or SLA in the absence or presence of FAK inhibitor (PP2; 10 μm) for 1 and 7 days were assessed for gene expressions of matricellular proteins, *CTGF* (A) and *THBS2* (B). Data represents mean fold gene expressions ± SD relative to day 1 PT without PP2 of three independent experiments in triplicates. *THBS2* mRNA levels of HGFs on PT and SLA at 1 and 7 days without PP2 (in a grey box) are shown at a higher scale. Data was analysed *via* two-way anova with bonferonni after test (*P < 0.05, **P < 0.001, ***P < 0.001, ****P < 0.0001). (C) Influence of FAK inhibition on fibronectin assembly and deposition on PT and SLA. Representative immunofluorescence images of HGFs cultured on PT or SLA in the absence or presence of PP2 (10 μm) at 1 day are shown. Cells were labelled for fibronectin (green) and nuclei (blue). PP2 added HGFs on PT resembled HGFs on SLA without the inhibitor.

As we observed that differential adhesion responses on PT and SLA led to altered gene expressions of ECM and adhesion molecules, we assessed how inhibition of FAK influences gene expression of *CTGF* and *THBS2*. PP2-treated HGFs were cultured on PT and SLA for 1 and 7 days. Consistent with the RT^2^ Profiler™ PCR array data, *CTGF* mRNA expression was significantly lower on SLA compared to PT at 1 day and 1 week (*P* < 0.05; Fig.9A). FAK inhibition led to significant decrease in *CTGF* on both PT (*P* < 0.001) and SLA (*P* < 0.01) at 1 day. There was no significant difference between PT with FAK inhibition and SLA without FAK inhibition (*P* > 0.05). *CTGF* also significantly decreased with time on both surfaces (PT: *P* < 0.0001; SLA: *P* < 0.01) in control conditions.

*THBS2* expression was significantly greater on SLA compared to PT at 1 day (*P* < 0.05) and 1 week (*P* < 0.01) (Fig.9B), consistent with the RT^2^ Profiler™ PCR array data. FAK inhibition significantly increased *THBS2* expression on both PT and SLA at 1 week (*P* < 0.0001). *THBS2* mRNA also increases over time on both surfaces (*P* < 0.0001) in control conditions.

To assess whether FAK inhibition affects matrix formation, we cultured HGFs with and without PP2 and assessed for fibronectin fibrillogenesis by using immunofluorescence at 1 day. As seen before, HGFs formed greater level of fibronectin fibrils on PT compared to SLA (Fig.9C). FAK inhibition decreased the level of fibronectin fibril assembly on the surfaces, where more diffuse fibronectin in the cytoplasm was observed. Fibronectin fibril organization on PT with FAK inhibition was similar in appearance on SLA without FAK inhibition.

## Discussion

Surface topography and roughness of implants has previously have shown to influence bone and soft-tissue integration 18,28. Research suggests that cell-substratum interactions determine tissue remodelling through integrin-mediated intracellular signalling, which is sensitive to substratum topography 17,29. However, perturbations in ECM remodelling and cell adhesion signalling are well known to contribute to the development of fibrosis and scarring 30. Implant surface topographies for promoting connective tissue remodelling and attachment while minimizing fibrous capsule formation is yet to be optimized, with the tissue structure around the transmucosal region often resembling the composition of a scar 3. Recent studies have suggested that rough topographies such as SLA can promote stable connective tissue formation 31–33, but as noted by Schwarz *et al*., the cellular response underlying soft-tissue healing at rough implant surfaces needs further investigation 33. The aim of this study was to assess and compare HGF response to PT and SLA surfaces.

Healthy gingival connective tissue has collagen fibres perpendicular to the tooth surface 34,35. However, the collagen fibres within the connective tissue around a dental implant typically are parallel to the surface 34–36. This results in insufficient tissue integration with the implant and insufficient blood supply to the site. Fibronectin, present in abundance in fibrotic lesions, is suggested to contribute to the excessive scarring observed in chronic fibrosis 37. Fibroblast attachment to fibronectin is known to be required for differentiation of fibroblasts into myofibroblasts; α-SMA level in cultured cells on fibronectin-coated plates was significantly higher compared to cells on other integrin-binding ECM surfaces 38. Here, we demonstrate a higher level of matrix fibronectin production and fibrillogenesis by HGFs on PT compared to SLA. Based on the number of tensin-containing fibrillar adhesion on PT, the topography of this surface appears to promote increased fibronectin fibrillogenesis and accumulation compared to the rougher SLA. We also observed greater myofibroblast differentiation indicated by cells with α-SMA containing stress-fibre networks on PT compared to SLA. Myofibroblast differentiation of HGFs observed on PT could be caused by excessive matrix fibronectin, as increased fibronectin accumulation is associated with increases in matrix stiffness 39. As a stiffer matrix environment has shown to be a prerequisite for fibrosis and induces fibroblast differentiation into myofibroblasts 5, cell attachment to smoother surfaces may alter mechanotransduction in the cells that increases myofibroblast differentiation. While only likely to be one potential factor in the overall gingival tissue response, PT topographies appear more likely to induce a fibrotic phenotype in HGFs around the abutment. Previous *in vivo* studies assessing connective tissue attachment to SLA and hydrophilic SLA demonstrated fibronectin deposition on the implant surface within 4 days, but no assessment of cell phenotype in relation to myofibroblast commitment was performed 32. Future studies should focus on whether myofibroblast differentiation is evident in close proximity to the implant surface.

We next assessed the potential molecular events leading to a myofibroblast phenotype on PT, but not on SLA. Upon adhesion, cells form focal contacts, which undergo process of maturity by recruiting structural and signalling proteins 27,40. FAs are mechanosensitive receptors that can mature according to the surrounding environment 41. It is known that nascent adhesions more frequently mature into FAs on stiff surfaces 41 and the size of a FA is an indication of its maturity 42. We show here that HGFs on PT formed significantly larger FAs compared to cells on SLA. This is consistent with a previous study, which showed that HGFs on SLA were forming less distinct vinculin-containing FAs, whereas distinct punctate FAs were observed on machined titanium 17. We also showed that the changes in adhesion size are concomitant with increased recruitment and phosphorylation of FAK. Phosphorylation of FAK is a known requirement for induction of α-SMA in response to TGF-β1 8. Thus, it appears that as PT surfaces permit formation of larger adhesion and higher levels of phosphorylation of FAK, adhesive signalling is activated which results in α-SMA expression and stress-fibre formation.

As FA size was smaller on SLA than PT, we next investigated how increased roughness affected adhesion formation. FAs are associated with integrin αvβ3, but as they mature, α5β1 receptors are recruited as FAs form fibrillar adhesions that can remodel the ECM 42. The translocation process is necessary for fibronectin fibrillogenesis and other matrix components 41,43. In our study, we performed immunocytochemistry to assess potential differences in localization of integrin subunits to FAs in HGFs cultured on PT and SLA. Our data show that on PT, integrin αvβ3 localized to FA, but on SLA integrin αvβ3 clustering was evident that did not co-localize with vinculin. Interestingly, neutralizing αvβ3 integrin led to a significant decrease in cell attachment to SLA but not on PT. Based on the presence of integrin αvβ3 clustering without vinculin, it is possible that there is more cycling of adhesion sites on SLA which we will investigate in the future. This is further supported by the recruitment of p-cortactin into adhesion sites on PT, but not SLA; p-cortactin stabilizes the F-actin cytoskeleton. Although both surfaces allow maturation of FAs into fibrillar adhesions, this was attenuated on SLA although HGFs are still able to form FN fibrils. Our finding supports that SLA slows induction and stabilization of mature focal and fibrillar adhesions while PT does not.

Changes in FA size have received much attention in relation to topographical modulation of cell behaviour. Mesenchymal stem cell to osteoblast differentiation has been correlated with increases in FA maturation 44,45. In contrast, topographical differentiation of human mesenchymal stem cells towards neurogenic and myogenic lineages was associated with smaller adhesion size on grooved substrata; these observations were correlated with FAK phosphorylation and of great significance, overexpression of FAK in these cells overruled any topographical effect 46. In the case of fibroblasts, our results are consistent with previous research*,* that a reduction in supermature or ‘fibrillar’ adhesions is associated with a reduced fibrotic response 47,48. We think it likely that it is the molecular components of the adhesions and subsequent activation of specific signal transduction pathways that govern the overall cell and tissue response, which is linked to size of adhesions and development of cell tension. By limiting the contact area between the fibroblasts and the titanium though particle blasting and acid-etching, the fibroblasts are not able to generate sufficient forces to induce fibroblast to myofibroblast differentiation.

As altered state of maturity and protein composition in FAs may contribute to altered cell signalling, tissue healing and remodelling 32, we next assessed how PT and SLA influence gene expression associated with ECM remodelling. On SLA surfaces, several genes were up-regulated including MMPs, matricellular proteins, integrins and matrix proteins, although the significance of all of these genes in gingival healing is unknown. At 24 hrs after seeding, tenascin-C was up-regulated on SLA compared to PT, which is expressed during normal gingival healing 49. Some of the highest fold increases were in MMPs, which are crucial regulators of ECM remodelling 50. Of the genes changed in HGFs by SLA, genes associated with ECM turnover were prominent; MMPs and TIMPs are intricately involved in maintaining the homoeostasis between synthesis and degradation of ECM components, but in healing and fibrosis the balance of these molecules is often altered. Previous studies demonstrate that MMPs and TIMPs can have both inhibitory and stimulatory roles depending on tissues and/or the type of MMPs 51. Our Profiler PCR array data demonstrated that several MMPs were up-regulated at 1 and 7 days, including MMP-7, -8, -9 and -16. MMP-8 mRNA levels was significantly increased by SLA compared to PT, almost a 4000-fold increase. MMP-8, also known as neutrophil collagenase, is significantly up-regulated in gingival healing around titanium implants in humans 52, as well as in skin healing 53 suggesting it is a prominent regulator of remodelling in these tissues. We observed up-regulation of TIMP3 expression on SLA compared to PT, but no difference of TIMP1 or TIMP2 expression on PT and SLA. This suggests that increased TIMP3 on SLA could be contributing to a reduced fibrotic response. The literature, together with our data suggest that up-regulation of MMP8 and TIMP3 on SLA may be critical players in reducing fibrotic responses. Future studies will investigate the role of all the up-regulated genes in HGF tissue development on PT and SLA, but in summary, our data support that the SLA topography induces genes associated with ECM remodelling in HGFs.

Our RT^2^ Profiler™ PCR array data show that of the genes that were tested in the array, CTGF or CCN2 was the only gene that was significantly down-regulated in cells on SLA compared to PT. CTGF is a pro-fibrotic matricellular protein that have been confirmed to be a critical player in fibrosis by modulating myofibroblast differentiation, and fibronectin production 54–56. CTGF is a potential player in peri-implant fibrosis and may be used for future therapeutic targets. Blocking FAK signalling attenuated higher expressions of CTGF on PT. Based on the role of adhesive signalling in fibrosis and our data demonstrating alteration in nascent FAs and mature FAs on SLA 42, it provides further evidence that alterations in substratum topography are a powerful tool to modulate matricellular protein gene expressions and matrix organization. This study further confirms the complexity of nascent *versus* mature FAs and their role in tissue remodelling.

Our study has revealed that limiting FA formation and stability leads to a reduced fibrotic response and increased tissue remodelling response in gingival fibroblasts *in vitro*. Application of the SLA surface to enhance gingival connective healing around implants has yet to be used clinically. Our study suggests that implant topographies that limit cell adhesion could be applied to prevent development of tissue fibrosis and scarring. This does, however, raise the question of how much cell adhesion is required to allow proper tissue formation and integration with the implant surface, which can only be addressed through *in vivo* studies. While we are not stating that SLA is the optimal substratum for abutments, our data show that topographical features which limit adhesion formation could be applied to not only implants, but any scaffold or device used in soft-tissue healing in which a fibrotic response is undesirable.

Cellular and molecular characterization of the response of HGFs on the smooth and rough titanium substratum is essential in the development of the optimal implant surface to manage the dental soft tissue-implant complex. Our study shows for the first time that HGFs form more mature FAs on PT compared to on SLA. The composition and stability of FAs seems to be a key determinant of gingival fibroblast response to altered substratum topographies. While HGFs are able to form matrix on both surfaces, our evidence shows fibrotic responses and adhesion-mediated fibrotic markers on PT but not on SLA. Our study suggests that fibrosis at the dental implant surface may be prevented by using an appropriate surface roughness on the abutment of the implant.

## Supporting Information

**Figure S1** Expression profile of 84 genes included in the ECM and Adhesion Molecules RT^2^ Profiler PCR Array of HGFs on PT and SLA.
