# Supplementary figures and images for: Regulation of matrix remodelling phenotype in gingival fibroblasts by substratum topography
Source: J Cell Mol Med. 2015 Mar 12;19(6):1183–96. doi: 10.1111/jcmm.12451 (PMC4459834; doi:10.1111/jcmm.12451)

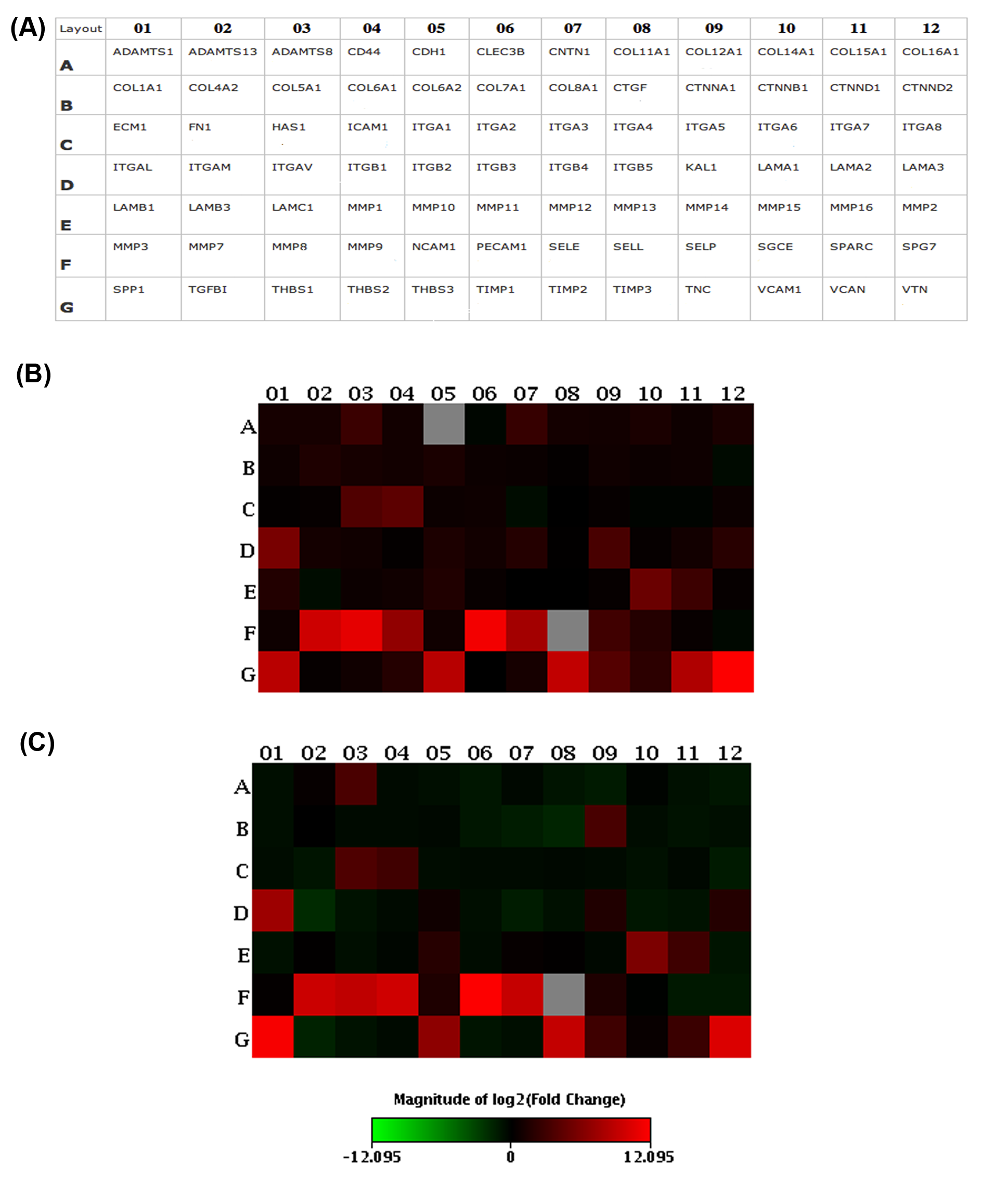

Supplement: Supplementary file 1 [file jcmm0019-1183-sd1.tif]
